# CRISPR/Cas9 gene editing: a novel strategy for fighting drug resistance in respiratory disorders

**DOI:** 10.1186/s12964-024-01713-8

**Published:** 2024-06-14

**Authors:** Bashdar Mahmud Hussen, Zana Baqi Najmadden, Snur Rasool Abdullah, Mohammed Fatih Rasul, Suhad A. Mustafa, Soudeh Ghafouri-Fard, Mohammad Taheri

**Affiliations:** 1 Department of Biomedical Sciences, College of Science, Cihan University-Erbil, Erbil, 44001 Kurdistan Region Iraq; 2 Department of Clinical Analysis, College of Pharmacy, Hawler Medical University, Kurdistan Region, Erbil, Iraq; 3 Research Center, University of Halabja, Halabja, 46018 Kurdistan region Iraq; 4 Medical Laboratory Science, College of Health Sciences, Lebanese French University, Kurdistan Region, Erbil, Iraq; 5 Department of Pharmaceutical Basic Science, Tishk International University, Kurdistan Region, Iraq; 6 General Directorate of Scientific Research Center, Salahaddin University-Erbil, Erbil, Kurdistan Region Iraq; 7 Department of Medical Genetics, Shahid Beheshti University of Medical Sciences, Tehran, Iran; 8 Institute of Human Genetics, Jena University Hospital, Jena, Germany

**Keywords:** Respiratory disease, CRISPR/ Cas9, Drug resistance

## Abstract

Respiratory disorders are among the conditions that affect the respiratory system. The healthcare sector faces challenges due to the emergence of drug resistance to prescribed medications for these illnesses. However, there is a technology called CRISPR/Cas9, which uses RNA to guide DNA targeting. This technology has revolutionized our ability to manipulate and visualize the genome, leading to advancements in research and treatment development. It can effectively reverse epigenetic alterations that contribute to drug resistance. Some studies focused on health have shown that targeting genes using CRISPR/Cas9 can be challenging when it comes to reducing drug resistance in patients with respiratory disorders. Nevertheless, it is important to acknowledge the limitations of this technology, such as off-target effects, immune system reactions to Cas9, and challenges associated with delivery methods. Despite these limitations, this review aims to provide knowledge about CRISPR/Cas9 genome editing tools and explore how they can help overcome resistance in patients with respiratory disorders. Additionally, this study discusses concerns related to applications of CRISPR and provides an overview of successful clinical trial studies.

## Background

Chronic respiratory disorders (CRDs) are a common set of conditions that mostly affect the lungs and airways. The World Health Organization (WHO) reports that 4 million people die from CRD each year, with around 90% of these fatalities happening in low- and middle-income countries [1]. They have a great impact on both individuals and society by increasing morbidity rates, mortality rates, suffering, and economic costs to humanity [2]. Long-term respiratory illnesses like lung cancer [3], pneumonia [4], and chronic obstructive pulmonary disease (COPD) [5] indeed pose significant challenges and can become more complex over time.

Generally, genetic inheritance, environmental variables, occupational exposure, or mutations are essential components in a category of severe chronic respiratory disorders [6, 7]. In most cases, a combination of many variables leads to the development of diseases such as interstitial pulmonary fibrosis, lung cancer, and COPD [8]. Despite the fact that the prevalence of these disorders is always on the rise, treatment options for respiratory illnesses are limited and largely include palliative care, which has a number of negative side effects and leads to low patient compliance.

Drug resistance is still the main obstacle preventing patients with respiratory disorders from being cured [9]. Several mechanisms have been identified to give rise to drug resistance in patients with respiratory disorders, including inhibition of drug influx, activation of drug efflux [10], alteration in the cell cycle, and alteration in binding sites [11]. Despite significant improvements in drug research, delivery systems, and modifications to current drugs, deaths from respiratory disorders are on the rise [12]. Various studies have shown that it is possible to target a wide range of human genes to limit drug resistance in human disorders by CRISPR/Cas9 [13, 14].

Clustered regularly interspaced short palindromic repeat (CRISPR)-associated protein-9 (Cas9) is a method for gene editing that consists of the DNA endonuclease Cas9 protein and a single guided RNA (sgRNA) [15]. The Cas9 is directed to the target DNA sequence by the sgRNA, which recognizes the genome’s corresponding complementary sequence [16]. When sgRNA and the target sequence are paired, Cas9 nuclease domains cause site-specific double-strand breaks around the proto-spacer adjacent motif (PAM), a region of sgRNA that is found near the 3′ end of sgRNA [17, 18]. After DSB induction, the Nonhomologous end joining (NHEJ) or HDR pathways allow cell repair machinery to repair and restore the genome [19].

With the help of the National Institutes of Health (NIH) recombinant DNA Advisory Committee, CRISPR/Cas9 technology is currently undergoing clinical trials. This technology enables to support cancer therapy by enlisting a patient’s T cells at the NIH [20, 21]. Furthermore, China granted ethical approval to a similar kind of clinical trial for gene therapy in human blood disorders [22]. These clinical studies could lead to the development of a useful therapeutic genome-editing system for the treatment of inherited or non-inherited genetic diseases in humans. Therefore, the potential use of CRISPR/Cas9 for targeting platforms in respiratory disorders therapy has only been partially explored. The long-term use of antibiotic therapies to treat CRDs such as bronchiectasis, severe asthma, cystic fibrosis, and chronic obstructive pulmonary disease can result in multidrug resistance. This study summarizes current knowledge of CRISPR/Cas9 gene editing and explores how it leads to overcoming therapeutic resistance in respiratory disorders. We also go through the main strategies used to solve CRISPR/Cas9’s limitations in clinical applications.

## CRISPR/Cas9 origin, structure, and mode of action

The acquired immune system, known as CRISPR/Cas9, was a defense mechanism against foreign invaders originally found in bacteria and archaea and used to defend themselves from viruses. In 1987, the odd repetitive DNA sequence that produced five copies of tandem repeats at the 3′ end of the *Escherichia coli* gene that codes for the alkaline phosphatase (AP) isoform converting enzyme (iAP) was initially identified by Ishino and his team [23]. In 1995, Mojica and colleagues used different bioinformatics techniques to find short sequences that were repeated and had similar structures in hundreds of microorganisms, and they named short regularly spaced repeats (SRSRs) [24]. This sequence was given the term “Clustered Regularly Interspaced Short Palindromic Repeats (CRISPR)” by Jansen and his team in 2002 [25]. Subsequent studies demonstrated the presence of a class of endonuclease-encoding CRISPR-associated protein (Cas) genes close to the CRISPR sequence and that bacterial Cas proteins cut the external DNA during phage invasion [26, 27]. Recently, an eukaryotic programmable RNA-guided endonuclease protein identified known as Fanzor [28].

The different CRISPR/Cas systems are categorized into two classes: class 1, which uses numerous effector molecules [29], and class 2, which uses just one effector molecule [30]. With fewer components, class 2 Cas systems, of which the Cas9 nuclease is the first and most extensively used, have drawn attention in engineering and development for use in gene editing [31, 32]. Consequently, most of the CRISPR/Cas proteins are derived from *Staphylococcus aureus* and *Streptococcus pyogenes* [33]. A prominent version of Cas9 that has been used has evolved in *Streptococcus pyogenes*, therefore referred to as Cas9 [34].

In general, CRISPR/Cas9 is composed of Cas nuclease protein and sgRNA [35]. The sgRNA is actually a combination of trans-activating CRISPR RNA (tracer RNA) and crRNAs [36]. Cas9 protein and DNA separation requires a ncRNA known as tracer RNA. While crRNA is a segment of RNA with the same complementary nucleotide sequence that is found within transposable elements in target DNA [37]. The sgRNA specifically binds to the target locus via its complementary 20-nucleotide sequence in the target DNA [38]. Moreover, a 2-6-nucleotide PAM motif must be present nearby for effective target recognition [39]. After sgRNA identifies the targets, then Cas9 brings it to the target locus for a double-strand break [40].

Moreover, Cas9 is an RNA-guided endonuclease that can find and cut target DNA whose sequence conforms to a template matching that of the guide [41]. Cas9 has two nuclease domains: From the RuvC-like domain and the HNH-like domain. The HNH-like and RuvC-like domains nick the target strand and non-complementary [42]. Cas9 exists in an unbounded state and has a recognition (REC) limb as well as a nuclease (NUC) one [43]. The REC-limb includes the guide RNA binding domain (REC1 and 2) along with a bridge helix, whereas the NUC-limb is composed of HNH, RuvC as well as PAM interaction regions [44]. To recognize site-specific DNA cleavage, Cas9 combines with sgRNA, a natural crRNA-tracrRNA complex, and other proteins to form an active DNA surveillance complex [45]. The tracrRNA is required for Cas9 recruitment, and the DNA target specificity is made possible by the 20-nt spacer region of the tracrRNA [46]. Nucleotide sequences within the spacer RNA of cRNA play a major role in target specificity [47].

**Fig. 1:**
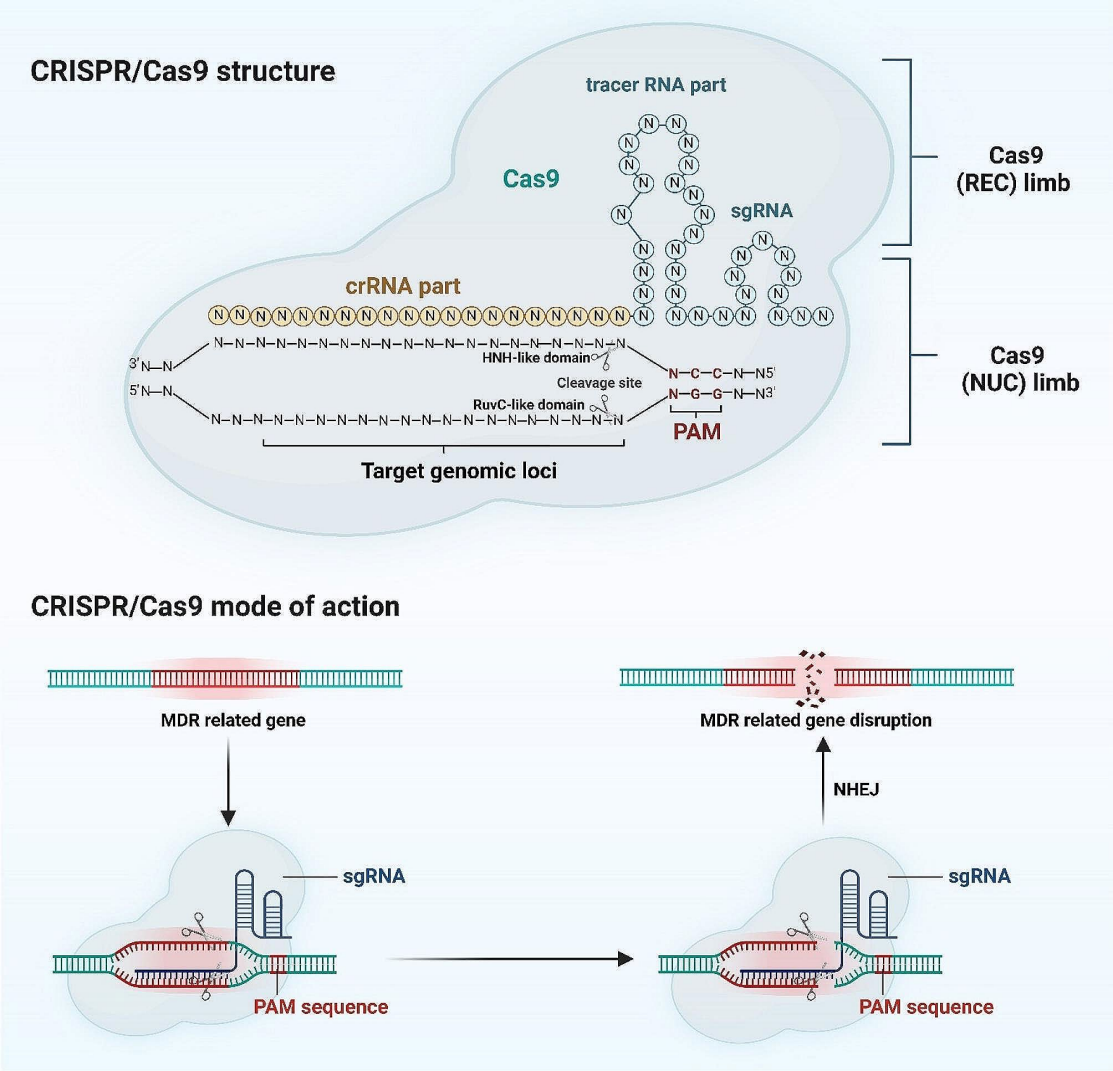
The schematic diagram shows the structure and mode of action of CRISPR/Cas9 for fighting drug resistance in respiratory disorders. The CRISPR/Cas9 system, consisting of the Cas9 endonuclease and single guide RNA (sgRNA), is depicted as targeting specific genomic sequences associated with drug resistance mechanisms in respiratory cells. Upon binding to its target sequence guided by sgRNA, Cas9 induces double-stranded breaks (DSBs) in the DNA and disrupts the multi-drug resistance (MDR)-related genes, thus leading to targeted genetic modifications that counteract drug resistance. The figure is made using BioRender software

After recognition, each single-stranded DNA (ssDNA) is cleaved, the HNH domains cut the DNA strand complementary to sgRNA, and the RuvC domain breaks the non-target strand, which is opposite to the complementary strand [48]. Following that damaged DNA repairs the breakthrough two mechanisms: firstly, homology-directed repair (HDR) is a precise repair mechanism that accurately repairs the DSB by using the homologous donor chromatid as a template, resulting in gene knock-in or point mutations; secondly, non-homologous directed repair (non-HDR) rejoin ends, which leads to indel formation, eventually achieving the goal of genetic changes (Fig. 1) [49]. Additionally, by simply alerting the nucleotide sequence of the sgRNA, the desired gene and occasionally several DNA targets can be targeted.

The competition between NHEJ and HDR is a critical aspect of CRISPR-based gene editing, profoundly impacting its applications. NHEJ, characterized by its error-prone nature, often leads to indels at repair sites, making it ideal for gene knockout studies [50]. In contrast, HDR offers precise editing capabilities but is less efficient and limited to specific cell cycle phases [51]. This competition significantly influences CRISPR applications; for instance, NHEJ predominates in many cell types, leading to functional gene disruptions, while HDR is preferred for precise mutations or gene insertions [52]. Strategies to selectively promote NHEJ are essential for tailored gene editing. These include timing CRISPR activity to favor NHEJ during active repair phases [49], and inhibiting NHEJ pathways to enhance HDR efficiency [53]. Understanding and manipulating this competition enable researchers to optimize CRISPR strategies for diverse gene editing needs, balancing precision with efficacy according to experimental requirements.

Recently, a method known as very fast CRISPR (vfCRISPR) has been developed, which enables the production of DNA-DSBs at the nanosecond and sub-micrometer levels, allowing for high-resolution studies of DNA repair in time, space, and genomic coordinates [54, 55]. Therefore, therapeutic genome editing still holds great promise for CRISPR-based methods.

Recent advancements in CRISPR/Cas9 gene editing have unlocked a myriad of cutting-edge applications for addressing drug resistance in bacterial infections [56, 57] as well as respiratory disorders. One significant area of progress lies in the development of CRISPR/Cas9-based therapies tailored for specific respiratory conditions such as lung cancer, pneumonia, and chronic obstructive pulmonary disease (COPD) [58]. These therapies are designed to target and modify disease-causing genetic mutations with unprecedented precision, offering personalized treatment options that were once thought to be beyond reach. Additionally, there have been remarkable strides in the field of delivery systems, with researchers focusing on enhancing the efficiency and specificity of CRISPR/Cas9 components’ delivery to lung tissues [59]. Advanced delivery methods, such as viral vectors and nanoparticles, are being explored to ensure targeted editing while minimizing off-target effects and immune responses [59]. Moreover, novel strategies are being devised to further mitigate off-target effects and immune reactions, including the development of modified Cas9 enzymes and delivery vehicles with improved safety profiles. These advancements collectively represent the current state-of-the-art in CRISPR/Cas9 gene editing, offering promising avenues for combating drug resistance and improving outcomes in respiratory disorders [60].

CRISPR/Cas9 technology has rapidly advanced since its initial discovery, leading to several modifications and improvements that have expanded its therapeutic utility [61] (Table 1). One of the key advancements and new modifications in CRISPR/Cas9 technology is base editing. Base editing systems have been developed to enable precise changes to individual DNA bases without creating double-strand breaks. This allows for targeted correction of genetic mutations associated with various diseases [62]. Moreover, prime editing is a newer CRISPR-based genome editing technique that enables more precise and efficient gene editing compared to traditional CRISPR/Cas9 methods. It can achieve targeted insertions, deletions, and substitutions in the genome without the need for donor DNA templates [63]. Another technology is the CRISPR interference (CRISPRi) and activation (CRISPRa), which allow for precise regulation of gene expression without altering the underlying DNA sequence; thus, they have potential therapeutic applications for controlling gene expression in disease states [64]. Additionally, enhanced specificity efforts have been made to improve the specificity of CRISPR/Cas9 systems to reduce off-target effects and increase safety in therapeutic applications. Various strategies, such as engineered Cas9 variants and guide RNA modifications, have been developed to enhance target specificity [65].

Overall, therapeutic applications of these advancements in CRISPR/Cas9 technology are vast and diverse, ranging from correcting genetic mutations underlying monogenic disorders to developing personalized cancer therapies.

**Table 1:** A comparison between the recently developed CRISPR/Cas9 modification and the traditional CRISPER/Cas9 that expands and enhances its therapeutic value

General aspects	Conventional CRISPER	New modification	Ref.
Off-targeting effect	Have an affinity for off-targeting effects	Responsible for reducing off-targeting effect	[66]
Efficiency	High efficiency	Their efficiency has been improved in targeting	[67]
Size of target DNA	Have the ability to target small fragments of DNA	Have the ability to target large fragments of DNA	[66]
Specificity	Limited specificities	Enhanced specificities	[68]
Editing range and speed	Targeted gene editing with a relatively fast editing	Expanded gene editing with enhanced editing speeds	[67]
Mechanism of DNA repair system	Depend on cellular repsir systems	Manipulates repair mechanism	[69]
Applications	A broad range of application	A diverse range of application	[70]
Delivery	Standard delivery systems	Improved delivery systems	[71]

## Drug resistance and respiratory diseases

When it comes to respiratory infections, drug resistance describes a pathogen’s capacity to resist the effects of drugs used to treat or manage the condition, such as bacteria or viruses. Bacterial pneumonia, tuberculosis (TB), and viral infections like COVID-19 and influenza are examples of respiratory diseases [72].

Drug-resistant strains of bacteria can arise from the abuse or overuse of antibiotics in cases of bacterial respiratory infections [73]. Bacteria can become resistant to the actions of conventional antibiotics by acquiring resistance genes or going through genetic alterations [74, 75]. The rapid spread of illnesses, including drug-resistant bacterial species, represents a serious threat to public health [76, 77]. In addition, the issue of drug-resistant TB is quite serious. Certain strains of the TB-causing bacterium have become resistant to first-line medications like rifampicin and isoniazid [78]. Remarkably, certain bacterial species undergo spontaneous genomic alterations while reproducing [79]. Mutations like this can alter antibiotics’ target sites, making them inefficient or useless against altered species. Further, antibiotics promote the survival of pathogens that have acquired mutations that make them resistant to the drugs [80]. Therefore, in drug-resistant populations, these strains have a better chance of surviving and spreading, eventually becoming the trend.

On the other hand, antiviral drugs are often used to treat respiratory viral illnesses like the flu or respiratory syncytial virus (RSV) [81]. Nonetheless, drug-resistant viruses can arise from mutations in viral genomes [82]. Due to this tendency, creating efficient antiviral medicines is challenging.

Mutations in the genetic material, genetic recombination of resistance genes, and the stimulation of inherent defensive systems are all mechanisms by which respiratory infections might acquire resistance to drugs, and they may cause respiratory diseases, including pneumonia and bronchitis [83]. These infections are caused by bacteria such as *Staphylococcus aureus* and *Streptococcus pneumoniae*, which have become resistant to medications such as penicillin and methicillin [84]. Further, they may make the treatment more challenging, increase the risk of treatment failure, and lengthen patients’ hospital stays. Therefore, drug resistance is a significant issue in respiratory diseases, affecting antibiotic efficacy and increasing the treatment complexity in respiratory infections.

## Application of CRISPR/Cas9 gene therapy for respiratory disorders

The discovery of CRISPR/Cas9, genome editing technology, has aroused great excitement, especially in treating respiratory illnesses, because of the promise of therapeutic correction of respiratory system endogenous gene mutations [85]. The first CRISPR-based treatment used in humans was for the treatment of resistant lung cancer. Initially, researchers took T-cells from three patients’ blood and modified them in the laboratory using CRISPR/Cas9 to eliminate genes that might impede the immune system’s ability to kill cancerous cells [86–88]. After that, the patients were reinjected with modified T-cells, and they found that modified T-cells put away malignant cells without any consequences [89].

**Fig. 2:**
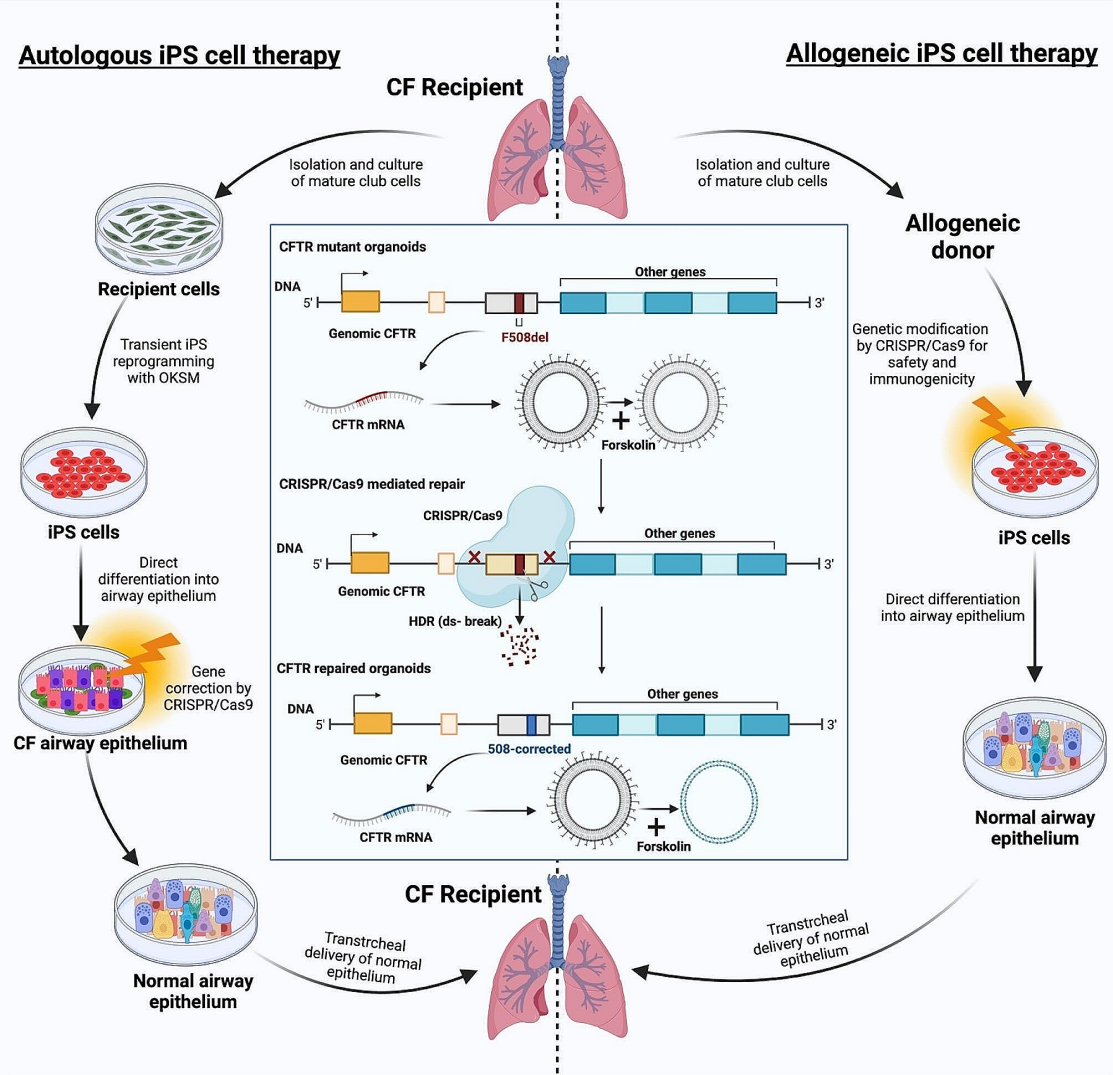
Illustration of induced pluripotent stem cell-based CRISPR/Cas9 technology. A patient with cystic fibrosis (CF) can undergo autologous iPS cell therapy, which involves isolating, expanding, and reprogramming their somatic cells to become induced pluripotent stem cells. These cells are then characterized into proximal airway epithelium, the genetic abnormality is fixed using CRISPR/cas9 technology to produce normal airway epithelium, and the cells are then implanted back into the recipient. The combination of allogeneic or autologous iPS cell therapy with CRISPR/Cas9 technology is a new area in stem cell therapy, allowing permanent changes in the DNA sequence precisely and personalized treatments for a wide variety of diseases and disorders. The figure is created using BioRender software

Respiratory disorders resulting from a single gene mutation, such as cystic fibrosis (CF), are good candidates for CRISPR-based treatments [90]. CF is an autosomal recessive life-limiting illness caused by a defect in the cystic fibrosis transmembrane conductance regulator (CFTR) protein encoded by the CFTR gene [91]. Small-molecule inhibitors are now unable to treat the majority of CF-related genes, which has led to the search for further therapeutic interventions [92]. Schwank et al. showed that the CRISPR system can repair CFTR dysfunction, investigated in a preclinical model employing intestinal stem cell-derived epithelial organoids [93]. In this model, forskolin-induced swelling of the organoid caused by an inflow of chloride and water indicated the presence of a functional CFTR protein. CRISPR repaired the CFTR locus by HDR intervention, and the corrected allele was expressed and fully worked in clonally grown intestinal organoids. CRISPR-HDR was then used to correct homozygous CFTR dysfunction in induced pluripotent stem cells (iPSC) taken from patients with CF [94] (Fig. 2). Subsequently, CRISPR-corrected iPSC developed into epithelial cells in the adult airway and exhibited restored CFTR performance. Similarly, Graham and his coworkers applied the CRISPR/Cas9 system to target multiple alleles in CF intestinal organoid cells. Base editing was used to correct the R785X mutation. Two other mutations, R553X and W1282X, were cured in clonal cell lines [95].

Respiratory problems can be treated using CRISPR through genetic interventions and epigenetic alterations. Point mutations in the endonuclease domains of Cas9 produce a mutant protein known as dead Cas9 endonuclease (dCas9), which lacks endonuclease activity [96]. The dCas9 directed epigenetic editing proteins to the SAM-pointed domain-containing Ets-like factor (SPDEF) promoter in human epithelial cells of the lung. SPDEF is thought to play a role in COPD-related mucus hypersecretion. This mechanism recruits transcriptional inhibitory complexes to the promoter, adds histone and DNA methylation, and inhibits SPDEF expression. Transcriptional suppression lasts across cell divisions, indicating that persistent phenotypic changes might occur without continuing to express CRISPR editing tools [97].

## CRISPR/Cas9 mediated genome editing and drug resistance in respiratory diseases

CRISPR/Cas9-based genome editing has become a transformative tool that offers precise and individualized methods to overcome drug resistance in respiratory disorders (Table 2). With the use of this cutting-edge technology, complex cellular and animal models that closely resemble respiratory diseases, including CF, COPD, and asthma, can be made [98, 99].

**Table 2:** The role of the CRISPR/Cas9 system mediated drug resistance in respiratory diseases

Types of Disease	Drug	CRISPR/Cas9-targeted gene and pathways	Animal study	Cell line	Delivery method	Total effect	Ref.
Tuberculosis	Isoniazid	KatG	-	-	Electroporation	The colony density of the mutant strain was not affected, while the wild strain was severely inhibited.	[100]
Pneumonia	Imipenem	bla_KPC−2_	-	-	Electroporation	Increase susceptibility to imipenem	[101]
Pulmonary aspergillosis	Itraconazole	167 mutation inAFUA_7G01960	-	-	Protoplast-polyethylene glycol	Increase MIC for itraconazole	[102]
Adenocarcinoma	PI3Kβ or PI3Kγ inhibitors	TP53, PI3K signal pathway	-	A549	Transfection	Knockout	[103]
Lung cancer	Cisplatin, Vinorelbine, Carboplatin	NRF2	Mice	A549	Transfection	Increasing chemosensitivity	[104]
	Cisplatin	ERCC1	-	H1299, H460, H522, H1703, H1650, H358, OV2008, C13	Transfection	Hypersensitivity of lung cancer cell lines	[105]
	Erlotinib	MDM4, PSMA6, PSMB6, ANAPC5, CDK1, Cell cycle processes or protein ubiquitination pathways	NOD/SCID/IL-2γ-receptor null (NSG) mice	NCI-H820, NCI-H1975	Transfection	Knockout	[106]
Lung adenocarcinoma	erlotinib/THZ1	MED1, CREBBP, EP300	Nu/Nu mice	PC9, HCC827	-	Knockout	[107]
	Selumetinib and Crizotinib	ROS1, MET, downstream PI3 K/Akt and MAPK signaling pathways	NOD/SCID gamma mice	HBEC, NIH-3T3	Transfection	Knockout	[108]
Lung squamous cell carcinoma	Combined FGFR1 and PLK1 inhibitor	FGFR1, FGFR pathway	Mice	H520, H1581, H1703, HCC95, PC-9, H1650, H1993, H2228, H226, H3122, H522, HFBN1, BEAS-2B	Transfection	Knockout	[109]
Non-small cell lung cancer	Paclitaxel	RSF1, NF-κB pathway	Nude mice	A549, H1299, HBE, H460, SPC	Transfection	Knockout Significantly increases apoptosis	[110]
	Cisplatin, Paclitaxel	Aurora-B-related p53 signaling pathway	-	A549	Transfection	Attenuating the p53-dependent DNA damage response	[111]
	Erlotinib	IGF1R, EGFR signaling pathways	-	HCC827	Transfection	Complete loss of IGF1R protein leads to decreased EGFR phosphorylation	[112]
	Osimertinib, AZ5104	EGFR, EGFR pathway	SCID mice	Cos-7, H2073	Electroporation	Silent mutation	[113]
	PI3K inhibitor BAY-1,082,439	P13K, PI3K signaling pathway	-	KB-3-1, MDR, NCl-H460	Transfection	Knockout	[114]
	Paclitaxel	KEAP1, AKT/ERK pathway, Wnt/β- catenin/TCF4 pathway	Nude mice	BEAS-2B, A549, H358, H1650, H460, H1975	Transfection	Knockout	[115]

*NFR2* nuclear factor erythroid 2-related factor 2, *Rsf-1* remodeling and spacing factor 1, *ERCC1* excision repair cross-complementation group 1, *IGF1R* insulin-like growth factor 1 receptor, *BlaKPC-2* beta-lactamase KPC-2, *EGFR* epidermal growth factor receptor, *MIC* minimal inhibitory concentration, *P53* tumor protein 53, *NSCLC* non-small cell lung cancer, *LUAD* lung adenocarcinoma, *LUSC* lung squamous cell carcinoma, *AD* adenocarcinoma, *LC* lung cancer, *FGFR1* fibroblast growth factor receptor 1, *PLK1* polo-like kinase 1, *GFR-TKI* growth factor receptor tyrosine kinase inhibitor, *NF-κB* nuclear factor-kappa B, *FGFR* fibroblast growth factor receptor, *PI3K* phosphoinositide 3-kinase, *MAPK* mitogen-activated protein kinase, *TCF4* transcription factor 4, *KEAP1* kelch-like ECH-associated protein 1, *MED1* mediator complex subunit 1, *Aurora-B* aurora kinase B, *TP53* tumor protein 53, *ROS1* proto-oncogene tyrosine-protein kinase ROS1, *MET* hepatocyte growth factor receptor, *MDM4* mouse double minute 4 homolog, *PSMA6* proteasome subunit alpha type-6, *PSMB6* proteasome subunit beta type-6, *ANAPC5* anaphase-promoting complex subunit 5, *CDK1* cyclin-dependent kinase 1.

### Targeting *Mycobacterium Tuberculosis* genome

Drug resistance in *Mycobacterium Tuberculosis* (Mtb) is increasing year after year, and this issue has become dangerous [116], especially after the COVID-19 outbreak [117]. The spread of multi-drug-resistant (MDR), extensively drug-resistant, and extremely drug-resistant strains [118], demands the development of new therapeutic approaches. Additionally, effective modification techniques are critical for identifying and characterizing pharmacological targets, as well as understanding mechanisms of resistance. However, genetic modification of mycobacteria is challenging because of the obvious risk to some species, their slow growth, and the high concentration of GC in their genomes [119].

Recently, the invention of the CRISPR/ Cas9 system has paved the way for the progress of numerous genetic engineering techniques for mycobacteria [120]. For instance, Feng et al. developed a CRISPR-guided DNA polymerase system for mutagenesis of targeted genomic loci in mycobacteria, which could significantly aid TB research and the development of antimicrobial resistance [121]. The system allows targeted nucleotide diversification within 2 months, making it efficient in slowly growing mycobacteria.

CRISPR/ Cas9 system also helps in understanding protein-drug interactions and can predict drug-resistant mutations in mycobacteria, potentially contributing to diagnostics and mapping of novel variants for drug assessment [121]. Meijers et al. used CRISPR/Cas9 to reactivate *Streptococcus thermophilus* CRISPR1-Cas9, thereby extending the genetic toolbox for mycobacteria. They demonstrated accurate and effective gene editing in mycobacterial species using a single plasmid containing the active Sth1Cas9. They transferred genes coding for aspartic proteases PecA to *M. tuberculosis* strains, creating frameshift mutations in 7 genes. They assessed the effectiveness of frameshift mutations produced by CRISPR/Cas9 in *M. tuberculosis* and found that frameshift mutations inactivate the target gene [100]. Moreover, Ding et al. developed a two-plasmid cytidine base editing system for genome editing in Mtb (MtbCBE), repressing homologous recombination and mismatch repair pathways and generating G: C to A: T base pair conversion, promising potential for mycobacterial physiology and anti-tuberculosis drug development [120].

Moreover, in Mtb, researchers have developed various CRISPR-based genome editing tools, including endogenous systems like the Type III-A CRISPR-Cas system [122]. This system, along with other CRISPR tools, presents exciting possibilities for tackling antibiotic resistance genes in Mtb strains. By leveraging the specificity and efficiency of these CRISPR systems, researchers can design crRNAs to precisely target and disrupt antibiotic resistance mechanisms, such as genes encoding drug efflux pumps or resistance enzymes [123]. This targeted approach holds significant potential for overcoming antibiotic resistance challenges and developing innovative strategies for managing drug-resistant tuberculosis infections.

In conclusion, immediate action is required due to the growing treatment resistance in *Mycobacterium tuberculosis* (Mtb) and the COVID-19 pandemic. New approaches to genetic editing, including the CRISPR/Cas9 system, provide hope for finding a solution. Research on tuberculosis and the creation of new drugs may benefit from CRISPR-guided mutagenesis and gene editing. These developments make it possible to comprehend the mechanisms underlying medication tolerance precisely and to develop innovative therapeutic approaches more easily to fight tuberculosis.

### Target pathogens in pneumonia

Community-acquired pneumonia (CAP) is an infectious lung parenchymal inflammation that develops outside the hospital. *Klebsiella pneumonia* [124], *Staphylococcus aureus* [125], *Pseudomonas aeruginosa* [126], *Haemophilus influenza* [127], and atypical bacteria, including *Chlamydia pneumonia* and *Mycoplasma pneumonia* are the most prevalent cause of pneumonia globally [128]. The development of innovative antimicrobial drugs or other alternative instruments to combat these infections depends on our ability to understand the mechanisms underlying the resistance of these bacteria. CRISPR/Cas9 is a promising tool for understanding and predicting multidrug-resistant pathogens, thereby revealing hidden or undiscovered resistance mechanisms [129].

The CRISPR/Cas system has multiple targets, enabling it to target many resistance genes simultaneously [130]. Hao et al. used the CRISPR/Cas9 system for *carbapenemase* gene editing in *Enterobacteriaceae*. They evaluated the effectiveness of *carbapenemase* gene curing for blaKPC-2, blaKPC-3, blaNDM-5, blaNDM-7, and blaOXA-48 in several species of *Enterobacteriaceae*, including *K. pneumonia* and *E. coli*. The results demonstrated the capability of the pasture platform to successfully eradicate blaKPC, blaNDM, and blaOXA-48-like gene variations in these strains with a cure frequency of > 94% [131]. Therefore, Zhan and co-workers proved that it was possible to genetically edit *K. pneumoniae* by using either the CRISPRc Cas9 system or a method based on lambda red recombination systems. So, they used the pCasKP-pSGKP and pBECKP systems to remove *carbapenemase* genes selectively, as well as extended-spectrum β-lactamase. They used pBECKP-spe to create a 4/12 potency blaSHV deletion mutant and inactivate two forms of carbapenems (blaKPC) and one form of cephamsics (blacTX), cleanly knocking out three plasmids at the same time. The results showed that knocking out the blaKPC-2 gene definitely made bacteria more sensitive to imipenem, but neither deletion nor inactivation affected drug susceptibility. This also confirms that the resistance of this strain of hyper-mucoviscous *K. pneumoniae* (KP CRE23) to carbapenems is mostly due to blaKPC-2, as expected [132]. D’Souza et al. used CRISPR/Cas9 to study AmpG permease gene deletion in *K. pneumonia*-YMC/2013/D, a carbapenem-resistant strain. Their results showed that an AmpG knockout increased carbapenem susceptibility, leading to a fourfold and two-fold reduction in imipenem and meropenem resistance, respectively [133].

### Target pathogens in pulmonary aspergillosis

The fungus Aspergillus causes a group of lung diseases known as pulmonary aspergillosis, which usually affects those with compromised immune systems or underlying respiratory disorders like asthma or COPD [134]. Since these infections can range in severity from mild allergic reactions to dangerous invasive disorders, treatment can be challenging, particularly when drug-resistant strains are involved. Recently, CRISPR/Cas9 has demonstrated potential in the management of lung conditions such as pulmonary aspergillosis [135].

*Aspergillus fumigatus*, *Aspergillus flavus*, *Aspergillus terreus*, and *Aspergillus niger* are the main pathogens associated with lung problems, while *Aspergillus fumigatus* is the most common pathogen [136]. For example, PCR fragments, Cas9 protein, and guide RNA were used to transfer a pan-azole-resistant *Aspergillus fumigatus* strain with cyp51A mutations into azole-resistant strains, revealing increased vulnerability by recombination utilizing CRISPR/Cas9 genome editing [137]. Similarly, Ballard et al. revealed that clinical isolates of *A. fumigatus* were exposed to various stress factors, revealing the complexity of adaptation processes. Genome editing systems like CRISPR/Cas9 have identified a specific in-host acquired SNP (AFUA_7G01960) that compromises azole therapy and is associated with non-cyp51A mediated antifungal resistance [102]. Their discovery reveals a novel mechanism of azole resistance in *A. fumigatus*.

### Asthma

Asthma is the most common chronic disease in children; patients have different clinical pictures, as mild-to-moderate asthmatics have T-helper cell type 2 (Th2), which are typically linked to eosinophilic airway inflammation, airway hyperresponsiveness (AHR), and mucus hypersecretion, and have a good response to corticosteroid treatment [138]. The second type of asthma patients have moderate to severe clinical pictures, comprise 25% of patients, are inadequate in responding to corticosteroids, and have monocyte and neutrophil airway inflammation together with a Th1, Th17 driven reaction [139].

Patients with severe steroid-resistant (SSR) asthma, which accounts for 50–80% of healthcare expenditure costs, presently have no effective treatments [140]. For instance, Martinon et al., in their study, used CRISPR/Cas9 to target NLRP3 in macrophages, disrupting the NLRP3 inflammasome. They developed a systemic delivery strategy using a cationic lipid-assisted nanoparticle (CLAN) to introduce mCas9/gNLRP3 into macrophages, reducing inflammatory conditions like septic shock, peritonitis, and type 2 diabetes [141].

### Lung cancer

A common and seriously fatal cancer that develops in lung cells is lung cancer. Non-small cell lung cancer (NSCLC) and small cell lung cancer (SCLC) are the two main forms that can be distinguished [142]. Innovative techniques like CRISPR/Cas9 gene editing have demonstrated potential in preventing medication resistance and enhancing lung cancer treatment outcomes. According to the findings of Hou et al., the p53 gene regulates the responsiveness of lung cancer cells to PI3K-specific inhibitors, PI3K-associated inhibitors, PI3K-non-related inhibitors, and protein-based stimuli. The deletion of p53 key regions with CRISPR/Cas9 altered p53’s structure and sequencing, leading to changes in PI3K subunit proteins or interactions [103]. Their study suggests that LC resistance can develop with dynamic mutation formations, highlighting the need for dynamic monitoring and drug resistance-specific targets.

**Fig. 3:**
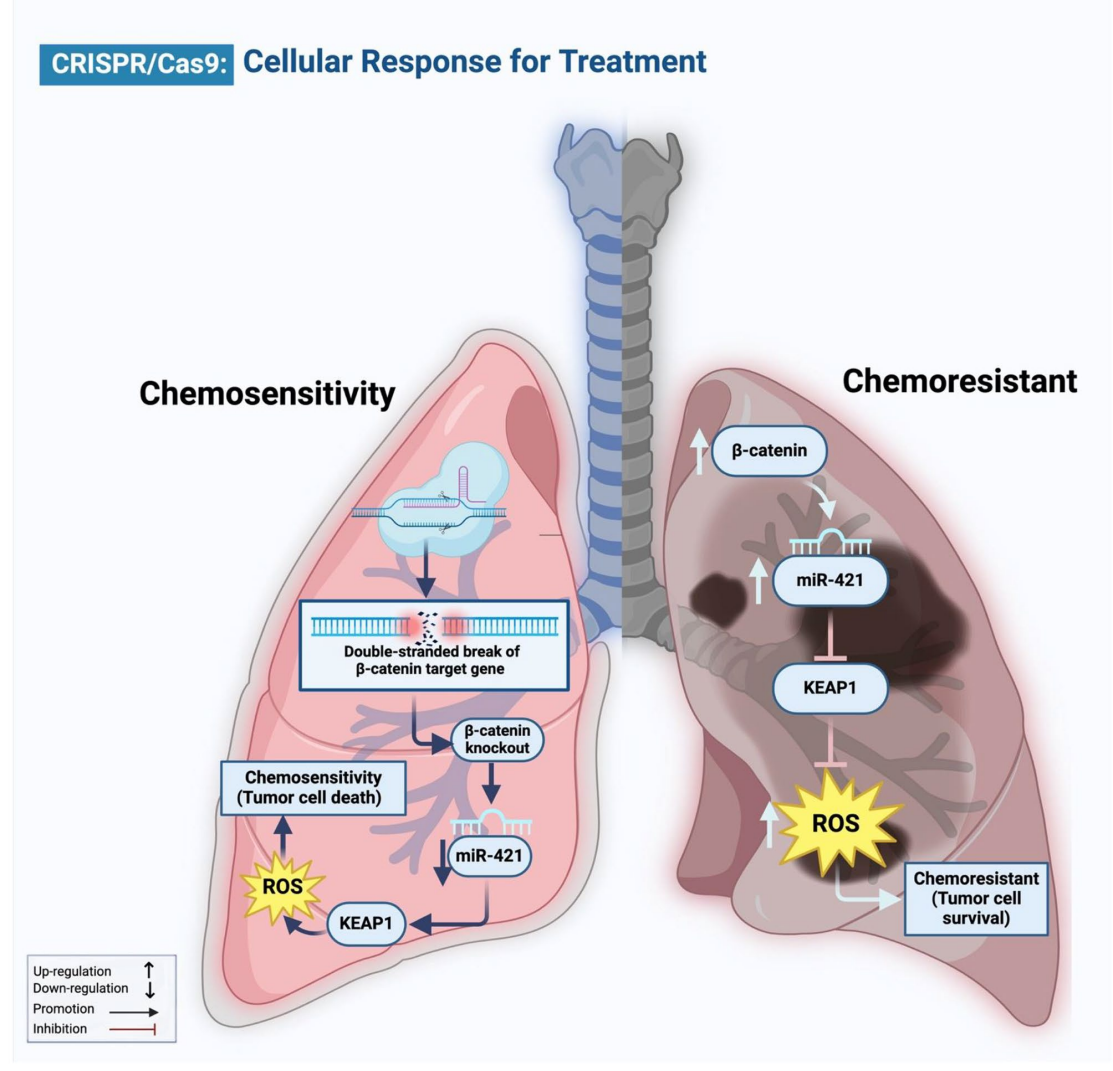
Schematic showing that CRISPR/Cas9 modulates the β-catenin-miR-421-KEAP1 pathway, which mediates tumor cell death by increasing chemosensitivity in LC. CRISPR/Cas9, comprising Cas9 protein and guide RNA (gRNA), targets specific DNA sequences, initiating the pathway with β-catenin activation and subsequent downregulation of miR-421. MiR-421 upregulates KEAP1, leading to ROS activation and enhanced antioxidant capacity. As a result, increases chemosensitivity, promoting tumor cell death in lung cancer cells. The figure is made using BioRender software

**Fig. 4:**
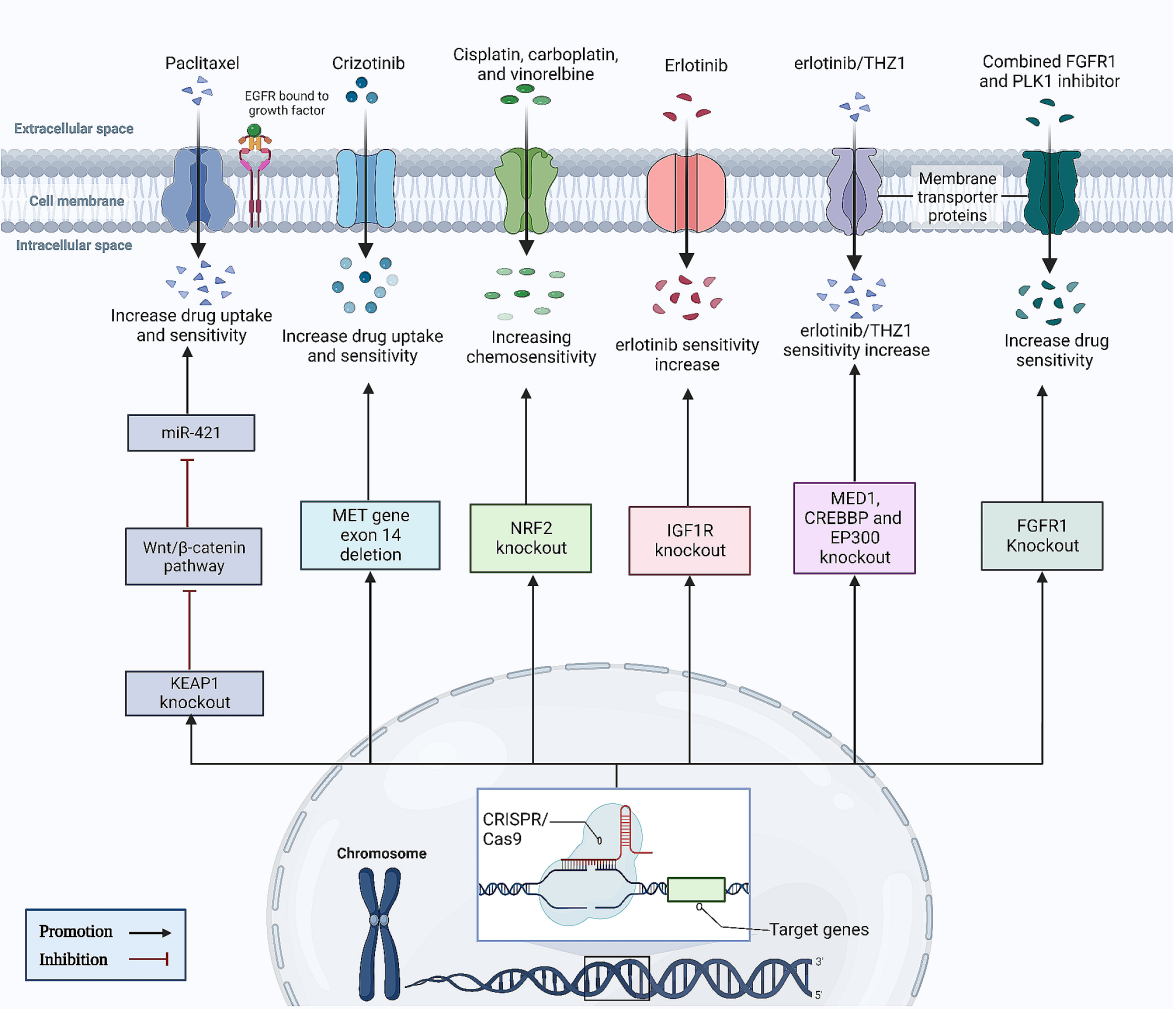
The schematic diagram represents the application of the CRISPR/Cas9 system for editing drug-resistant genes and identifying potential targets in respiratory diseases drug-resistant. The figure is made using BioRender software

Similarly, Chen et al. confirmed that CRISPR/Cas9 deleted the RSF1 gene when combined with paclitaxel, reducing cell migration and proliferation, subsequently increasing apoptosis in H460 and H1299 cells, and causing cell-cycle arrest in G1. RSF1 deletion significantly increased paclitaxel sensitivity and lightened the load (P b0.01) and volume (P b0.05) of the transplanted tumor in the xenograft mice model of LC using H460 cells [110]. Likewise, Duan and his team, in order to examine the connection between miR-421 and β-catenin, deleted β-catenin using CRISPR/Cas9 in A549 cells. They observed that knockout of β-catenin decreased miR-421 expression in A549 cells and increased Paclitaxel drug uptake in NSCLC patients [115] (Fig. 3). Further, Togashi et al. used the CRISPR/Cas9 technology to generate an in vitro model with MET exon 14 deletion, and they examined the phenotype that resulted, including its susceptibility to a MET inhibitor. As a result, they observed that MET exon 14 deletion enhanced sensitivity to the Crizotinib drug, a MET inhibitor [108]. Further, in A549 cell lines, Bialk et al. generate an NRF2-knockout clonal by a CRISPR-directed gene-editing technique. Based on their investigation, they concluded that, even in LC patients with the most stressful environmental conditions, the genes responsible for the efflux of anticancer drugs would not be activated, making cells with this gene knockout more susceptible to chemotherapeutic agents like vinorelbine, carboplatin, and cisplatin [104]. Moreover, according to Hussmann et al., by utilizing CRISPR/Cas9 to induce a genetic deletion, an IGF1R knock-out HCC827 cell line was produced. They discovered that in HCC827 NSCLC cells, IGF1R depletion promotes MET-amplification as a mechanism of acquired resistance to erlotinib [112]. Additionally, in EGFR-dependent LC PC9 cells receiving combination therapy with erlotinib + THZ1 (CDK7/12 inhibitor), Terai and colleagues conducted a genome-wide CRISPR/Cas9 enhancer/suppressor screen. This combination has been previously demonstrated to suppress drug-tolerant cells. As a result, Erlotinib/THZ1 synergy was predicted to be increased by the inhibition of several genes linked to transcriptional complexes such as (EP300, CREBBP, and MED1) genes [107]. Furthermore, Yang and colleagues employed FGFR inhibitor-treated FGFR1-amplified lung cancer cells to conduct kinome-wide CRISPR/Cas9 loss-of-function tests. These screens revealed PLK1 to be a strong synthetic lethal target that, with FGFR1 inhibition, overrides DNA damage and cell-cycle arrest, hence mediating a resistance mechanism. Through the stimulation of the γH2AX–CHK–E2F1 axis, the genetic and pharmacological antagonists of PLK1 in conjunction with FGFR inhibitor therapy synergized to boost antiproliferative effects and caused cancer cell death in vitro and in vivo [109] (Fig. 4). Therefore, CRISPR/Cas9 makes a revolutionary step in treating resistant carcinomas of the lung.

### Influenza virus infection

Influenza virus (IV) infections or acute viral respiratory infections are highly contagious and can cause pandemics, epidemics, and outbreaks [143]. The WHO estimates that each year, seasonal influenza virus (seasonal IVs), comprising the H1N1 and H3N2 IAVs and influenza B viruses, cause between 3 and 5 million severe cases and 290,000–650,000 fatalities worldwide [144]. The speed with influenza viruses might develop resistance, which limits the effectiveness of available antiviral treatments [145]. Drug resistance has been a significant issue affecting antiviral medications’ ability to combat influenza. A large amount of resistance has developed to the two main groups of antiviral medications, neuraminidase inhibitors (NAIs) and adamantine, both of which are frequently prescribed to treat influenza.

The adamantanes (M2 ion channel inhibitors) are no longer in use because of widespread influenza virus resistance [146]; instead of treating influenza virus infections, NAIs like oseltamivir are now the sole extensively used alternative [147]. However, after prolonged therapy, oseltamivir resistance has also been noted [148]. Before specialized vaccines are available, innovative antiviral methods are crucial for providing the first line of defense against emerging epidemics and pandemics [149]. The abuse of oseltamivir has led to evolve new drug-resistant mutant viruses and threatens public health.; like the seasonal H1N1 influenza virus that developed oseltamivir resistance in 2007–2008 without the use of NAIs [150], this is rising notably among patients admitted to (ICU). As an illustration, Behillil et al. did a French multicenter observational cohort study; they found that 23% of patients develop oseltamivir resistance, which is associated with increases in death and morbidity [151]. Moon et al. demonstrated CRISPR/Cas9’s ability to detect drug-resistant strains by adding viral lysates and PAMmer to dCas9/sgRNA-attached well plates and using a horseradish peroxidase reaction to identify viruses [152]. This is hopeful, as CRISPR/Cas9 can be used to detect drug-resistant viruses.

Furthermore, Favipiravir is a brand-new medication that may be used to treat influenza [153], but resistance may occur, specifically in the case of a new pandemic. This drug attacks the viral RNA-dependent RNA polymerase (RdRP). All strains of influenza A and B are susceptible to the Favipiravir. Likewise, Goldhill et al. showed that Favipiravir resistance could develop in Influenza A/England/195/2009 (Eng195), early isolated from the 2009 H1N1 pandemic. They demonstrated that for resistance to emerge, two mutations-P653L in PA and K229R in PB1- were required [145].

The ineffectiveness of adamantanes and worries about oseltamivir resistance impede treatment attempts due to resistance to antiviral drugs. Novel approaches, such as CRISPR/Cas9 tool for drug-resistant gene identification, show promise. Novel medications like favipiravir have promise, but monitoring is required to avoid resistance. To handle the changing problems that influenza viruses present, more research is necessary.

## Clinical trials of CRISPR/Cas9 gene editing for respiratory drug resistance

Scientists have continually used gene editing technology to understand the function of human genome, and it has clinical applications in numerous inherited genetic diseases and drug resistance [154]. Clinical trials may employ modified CRISPR/Cas9 gene editing techniques that are both safe and effective. It has become possible to create a CRISPR-related transposase that can both cut DNA and carry out precise editing [155]. Various CRISPR frameworks, including chemically and light-activated Cas9, have been explored for use in in vivo gene therapy [71, 156]. Additionally, small CRISPR modifiers have been the focus of recent research [157].

Interestingly, targeted genetic changes linked to resistance mechanisms have been identified by CRISPR/Cas9 gene editing, which shows potential for treating drug resistance in respiratory diseases [158]. Clinical trials are underway to evaluate the safety and efficacy of CRISPR/Cas9 gene editing in overcoming drug resistance in respiratory disorders. For instance, a clinical study looking into the application of CRISPR/Cas9 to target mutations in the CFTR gene, which causes cystic fibrosis. By correcting these mutations, researchers hope to restore the proper function of the CFTR protein and improve outcomes for patients with cystic fibrosis who have developed resistance to existing treatments [159].

In another clinical trial study (NCT02793856), non-small cell lung cancer was tried to be cured with CRISPR-engineered patient-derived T cells targeting the PD-1 gene. To disrupt PD-1 gene-associated exons, plasmids encoding Cas9 and sgRNA were electroporated into patient-derived T cells [21]. Further, in a group of patients with advanced lung cancer, the safety and viability of CRISPR-Cas9 gene-edited T-cell therapy directed against the PD-1 gene was proven. This strategy showed little off-target consequences and therapeutically is viable.

Additionally, KRAS mutations are commonly found in various types of cancer, including NSCLC. One specific mutation, KRASG12C, has been identified as a potential target for therapy in NSCLC [160]. Several inhibitors targeting this mutation have shown promising results in preclinical studies and early-phase clinical trials.

The first KRAS (G12C) small molecule inhibitor to enter clinical trials is AMG510 (NCT03600883), which binds to Cys12 in the inducible S-IIP precisely and irreversibly to lock the KRAS (G12C) protein in an inactive state [161, 162]. However, the development of acquired resistance to KRAS (G12C) inhibition is a significant challenge in the treatment of NSCLC. According to a study by Liu et al., liquid nitrogen-treated (LNT) cells are being used to deliver CRISPR-Cas9 nanoparticles for treating KRAS-mutant NSCLC [163]. In addition, a phase 3 NCT04303780 trial comparing AMG 510 with docetaxel in patients with NSCLC who had a KRAS p. G12c mutation used a combination therapy. Notably, safety issues forced Eli Lilly to withdraw their first KRAS (G12C) inhibitor, LY3499446, but they also revealed preclinical results for another inhibitor, LY3537982, at the 2021 AACR meeting [164].

This innovative method preserves the stability and viability of cells used for the delivery of CRISPR-Cas9 components, allowing for efficient targeting and correction of KRAS mutations. Further, it offers a personalized treatment option for KRAS-mutant NSCLC, improving clinical outcomes and combating this aggressive lung cancer.

Overall, the use of CRISPR/Cas9 to treat respiratory disorders and overcome drug resistance is clearly of interest; nevertheless, the number of studies and details on ongoing clinical trials are limited recently. Further research would be needed to find specific trials and their current status.

## Strategies to control the main barriers of CRISPR/Cas9 use as a replacement for drug-resistance therapy

CRISPR/Cas9 shows considerable promise as a potential replacement in human disorders for drug-resistance therapy. CRISPR/Cas9 systems have advantages and disadvantages when applied to respiratory problems. In general, they have precision [165], efficiency [166], versatility [167], and the potential for personalized medicine [168] advantages. However, there are various barriers in the way of its extensive clinical application. CRISPR-based gene editing techniques face several challenges, including immunological responses, off-target effects, and delivery issues. Here are some approaches to advancing therapeutic CRISPR/Cas9 use and managing these obstacles:

### Immune response to Cas9

The growth of immune responses against the Cas9 protein is a major impediment to the use of CRISPR/Cas9 in human treatment, which can limit the efficiency of the therapy and pose risks to safety [169]. Researchers are investigating numerous approaches to control this barrier. On the one hand, creating new Cas9 variants with lower immunogenicity may open a new door to overcome this issue [170]. Further, concurrent immunosuppressive treatments may be used to reduce immunological reactions against Cas9 [171]. Patients’ immunological reactions can be tracked and studied to identify future problems and provide individualized treatment plans. Moreover, to lessen immunogenicity, protein editing or the use of non-human Cas9 orthologs may be options to consider [172]. However, to ensure the long-term efficacy and security of CRISPR/Cas9 as an alternative for drug-resistance therapy in human illnesses, the immunological response to Cas9 must be addressed.

On the other hand, early-life gene editing, a controversial and ground-breaking area of genetic research, entails the modification of a person’s DNA at an early stage of development, frequently before or soon after birth. This strategy primarily makes use of the ground-breaking CRISPR/Cas9 technology, which enables precise and targeted alterations of particular genes [47]. Even prenatally, many disorders can be diagnosed, and management of these diseases early in life may save the lives of thousands all over the world. For example, CRISPR/Cas9 is successfully applied in several diseases like cystic fibrosis, mucopolysaccharidosis type IVA, and sickle cell anemia during childhood, which leads to treating the disease by CRISPR/Cas9 before the patient gets immunity to Cas9 [173]. Thus, Human CRISPR/Cas9 therapy is hampered by the immune system’s reaction to Cas9; nevertheless, scientists are investigating ways to overcome this problem, including creating Cas9 variations that are less immunogenic and employing immunosuppressive therapies. For instance, to overcome immunity to Cas9, one strategy is to deliver Cas9 mRNA into cells instead of the Cas9 protein [174, 175]. This approach can bypass pre-existing immunity to the Cas9 protein, thereby enhancing the effectiveness of CRISPR-based genome editing. When Cas9 mRNA is delivered into cells, it can be translated into the Cas9 protein within the cellular environment [176]. This circumvents the need for direct introduction of the Cas9 protein, which may trigger immune responses in some individuals. However, to maximize the effectiveness and safety of CRISPR/Cas9-based treatments in treating human diseases, more research is essential.

### Off-targeting

In the field of genetic editing, off-targeting by CRISPR/Cas9 is a major challenge. A targeted modification can be made to the DNA of an organism using the potent and precise gene-editing technology CRISPR/Cas9 [177]. Off-targeting occurs when the Cas9 protein binds to and cuts DNA at a location that is similar to the intended target but not the same as it [178, 179]. There is a possibility that this could lead to alterations in locations within the genome, potentially causing unforeseen and adverse consequences [180]. Other variables in the machine that can influence this probability include the specificity of sgRNA, quantity and concentration of Cas9, and duration exposure to editing machinery [181].

**Fig. 5:**
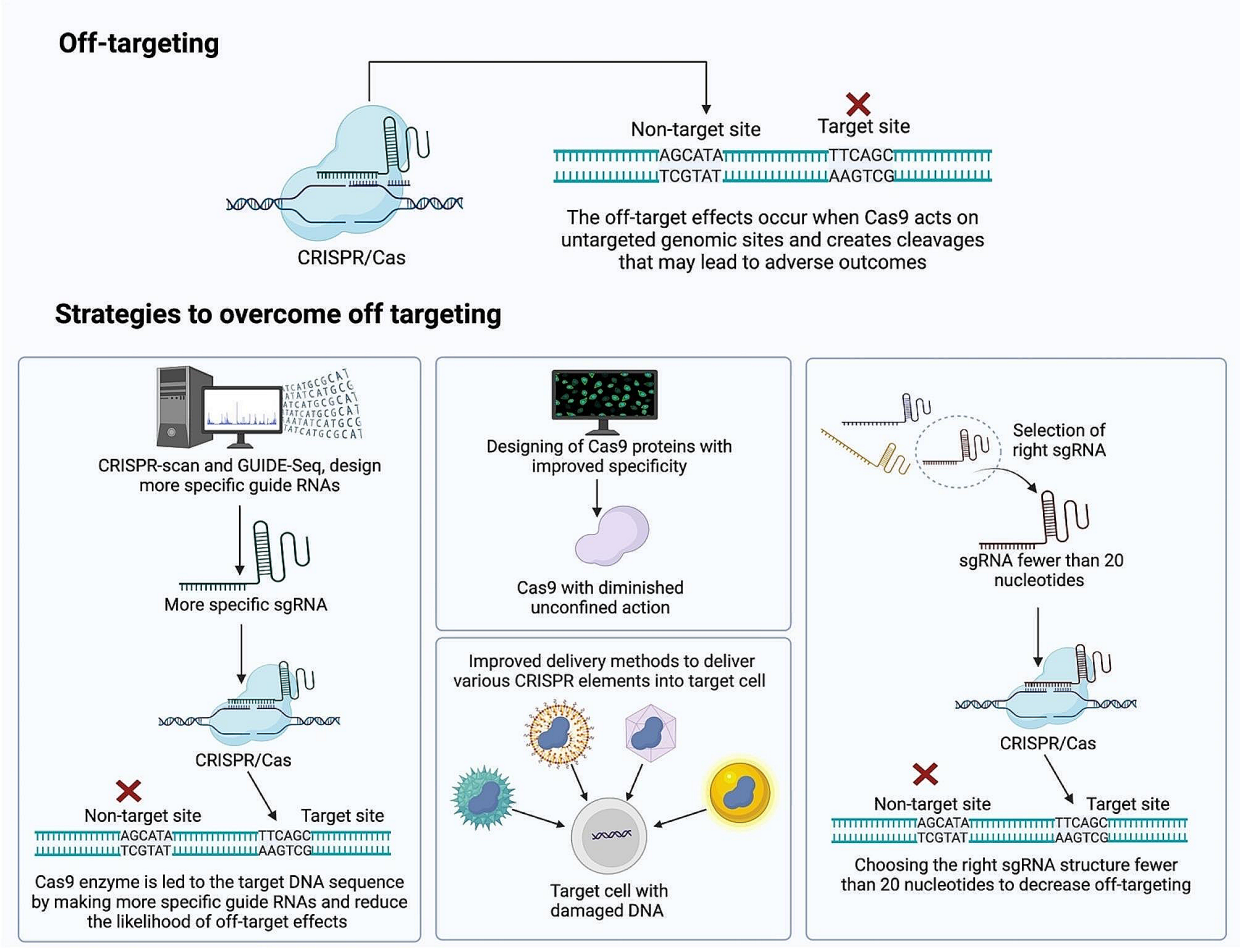
The schematic illustration represents the CRISPR/Cas9 system’s off-targeting challenges, as well as main important strategies to overcome these challenges. The figure is made using BioRender software

By establishing more precise target-guide RNAs and by creating Cas9 with diminished unconfined action over the chromosome, researchers have achieved significant gains in improving CRISPR/Cas9 accuracy [177]. Scientists can employ a variety of tools, such as rigorous screening for potential collateral sites, to minimize the risk of off-targeting [182], Cas9 mutants with greater specificity [183], and improving methods to deliver various CRISPR elements into target cells [184]. Further, bioinformatics methods assist in enhancing the design of sgRNAs, identifying the location for editing across the genome, and minimizing the probability of off-target effects. Choosing the right sgRNA structure is crucial to decrease the possibility of off-targeting (Fig. 5). For instance, multiple studies have established a clear association between sgRNA length and the number of off-targeting [185, 186]; for example, sgRNAs of fewer than 20 nucleotides have a considerable act on decreasing off-targeting [187], and sometimes the length of fewer than 15 nucleotides loses its specificity [188].

In addition, defending mechanisms in the form of naturally occurring host proteins can resist the CRISPR/Cas system. New opportunities for gene editing and bioengineering have emerged as a result of the discovery of anti-CRISPR proteins [189]. Researchers have utilized these proteins’ power to increase the accuracy and control of CRISPR-based genome editing methods. Researchers can control the time and scope of gene editing by including anti-CRISPR proteins alongside the CRISPR-Cas system, minimizing off-target effects and improving the accuracy and safety of genetic alterations.

### Delivery challenges

There is also an issue of how to get the CRISPR system to the right place at the right time in the right sequence, and this presents its own set of challenges. Various methods, including physical, viral, and extracellular vesicle-based system procedures and techniques, are used in CRISPR/Cas9 technology [190].

Cas9 and sgRNAs can be physically injected into cells using a microscope and a needle technique called microinjection. Due to its limited cloning ability, the molecular weight of Cas9 is typically a problem in viral vector-mediated delivery; however, in microinjection, this is not a problem because the needle pierces through the cell membrane to deliver the payloads directly into the nucleus [191]. Furthermore, the precise dosage of payloads in cells is made possible by manual injection. Nevertheless, the technique of microinjection has low throughput due to its arduous and technically complex nature. Furthermore, this technique cannot be used for in vivo patient work because it requires a microscope for injection [192]. In fact, animal zygotes are the primary source of microinjection applications, which create transgenic animal models [193].

One common kind of physical method of CRISPR/Cas9 delivery is electroporation. It uses electrical current pulses to temporarily open pores in cell membranes, allowing cargo to be delivered into cells [194]. Because electroporation effectively transfers payloads into a broad range of cell types, it is frequently utilized in both in vivo and in vitro gene editing [195].

**Fig. 6:**
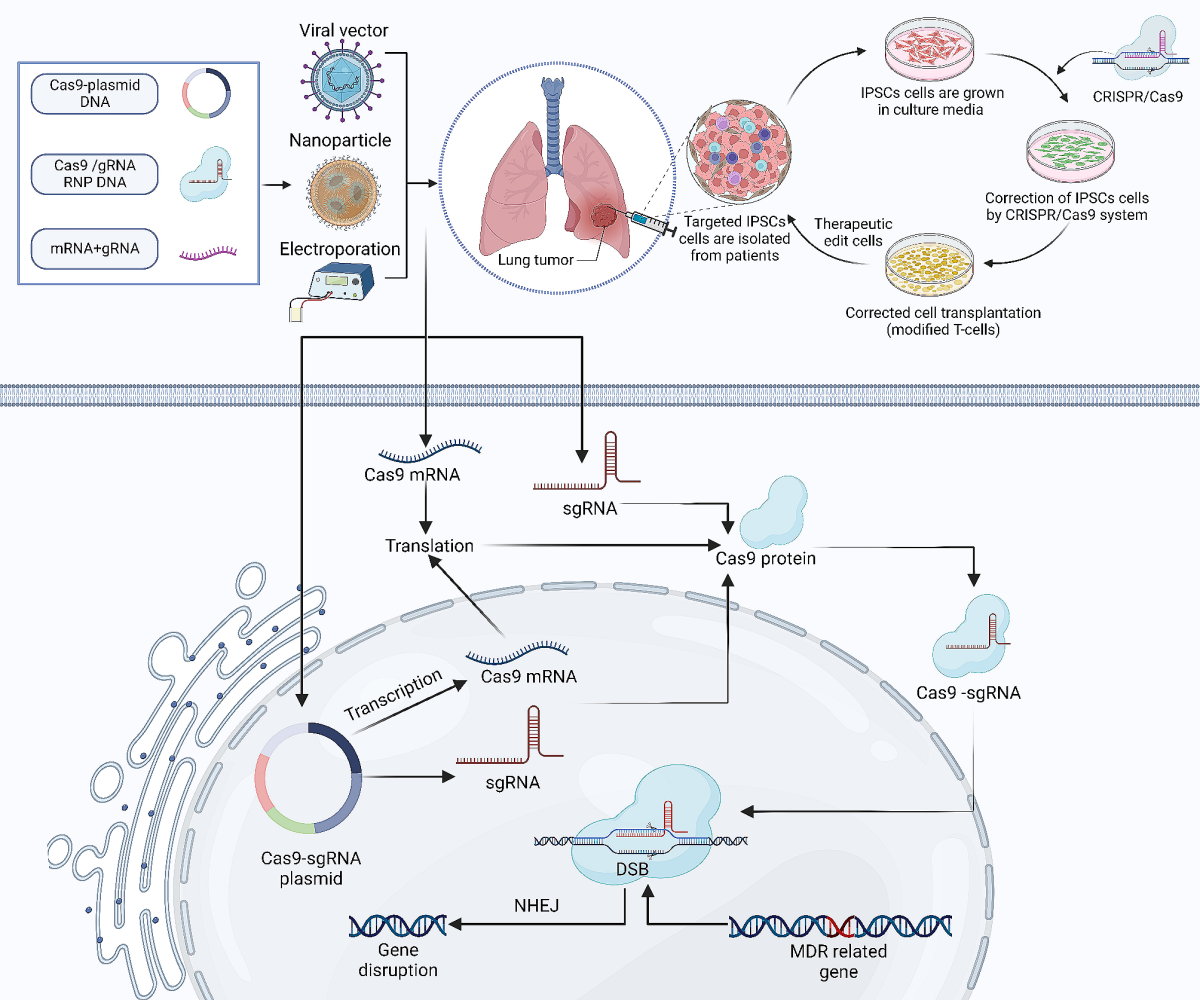
Schematic representation illustrating the CRISPR/Cas9 system’s targeting of an MDR-related gene and correction of IPSCs cells. The CRISPR/Cas9 system is delivered into cells via a variety of techniques, including electroporation, viral vectors, and nanoparticles. After the transcription and translation of Cas9 mRNA, the Cas9 protein is Combined with sgRNA. This complex then specifically targets genes associated with multidrug resistance (MDR). Induces a break in the DNA strand. The figure is made using BioRender software

Viral vectors like an adeno-associated virus (AAV) composed of lentivirus and adenovirus have been used safely in many studies [196]; only a few limitations present, like immunological reactions and the small size of the packaging [197], that can be solved by using multiple vectors to transport the system. Using non-viral vectors like lipid nanoparticles and inorganic nanoparticles will reduce off-target effects because they are effective and safe delivery methods and are more precise in targeting, which have less immunogenicity and exposure to nucleases [174] (Fig. 6).

Further, a new strategy for improvement in delivery methods is using extracellular vesicle-mediated delivery of Cas9 ribonucleoprotein (RNP) complex like recombinant CRISPR-Cpf1 Ribonucleoprotein (CRISPR-Cpf1-RNP) to decrease the chance of off-target effect, the efficacy of the system will be much more in comparison with using target delivery systems [190].

## Discussion

Human health continues to be greatly impacted by respiratory conditions such as influenza infection, acute tracheal bronchitis, pneumonia, tuberculosis, asthma, chronic obstructive pulmonary disease, lung cancer, and nasopharyngeal carcinoma [198]. Environmental and socioeconomic variables can have an impact on lung and respiratory diseases, but genetic or epigenetic factors also play a significant role in many of these major respiratory problems [199].

In the field of molecular biology, genome editing has become a ground-breaking field. The CRISPR-Cas9 system, which combines short palindromic repeats with regular spacing between them, is the leading technique in this field and has garnered significant interest [200]. The Cas9 protein and the sgRNA are the two main parts of the CRISPR-Cas9 system. As a molecular scissor, Cas9 cleaves DNA at specific target places, guided by the target DNA sequence aligned with the gRNA [201]. CRISPR-extraordinary Cas9’s precision and versatility have brought about a revolution in genome editing. Because of its precision and versatility of use, this molecular machinery, which was first developed from bacterial adaptive immune systems, has been used to modify the genomes of many different creatures, including humans [187].

Targeted gene knockouts may be quickly created in cells and animals using CRISPR editing, which is very helpful for understanding the mechanics of respiratory physiology and illness. Thus far, the role of genes involved in surfactant generation, senescence of epithelial cells in COPD, and inflammation and fibrosis in nasal and lung epithelial cells has been validated by the use of CRISPR-edited mutant cell lines and animals [198]. Just like Heyza et al., the study revealed that ERCC1 loss hypersensitizes cells to cisplatin when wild-type p53 is retained, while p53 mutation/null cell lines show modest sensitivity. Disrupting p53 by CRISPR-Cas9 reduces apoptosis and increases viability, indicating cisplatin tolerance in ERCC1 deficiency [105]. Similarly, Yu et al. confirmed that knocked out of Aurora Kinase B (AURKB) by using the CRISPR/Cas9 tool restored p53 expression and improved the cells’ susceptibility to cisplatin and paclitaxel [111].

Nonetheless, a number of restrictions and difficulties still exist and ought to be removed. Future research should focus on a few limitations, such as the consequences of Cas9 nuclease expression over an extended period in vivo, the durability of the targeted gene abundance, and potential immune responses to the nuclease and specific protein. Prior to the application of CRISPR/Cas9 for the correction of human disorders, efforts were undertaken to improve and optimize editing efficacy, minimize off-targets, and create cutting-edge tools for accurately delivering CRISPR/Cas9 modules to the target tissue for gene editing [202].

This approach has some inherent limitations that affect this investigation. First of all, there is a serious risk of off-target consequences, which could result in unwanted genetic changes. It’s still difficult to ensure high specificity while reducing off-target impacts. The respiratory system’s target cells are difficult to reach with CRISPR/Cas9 components, necessitating precise and effective delivery methods. Furthermore, there are hazards associated with the immunogenicity of CRISPR/Cas9 components and possible immune responses, especially with repeated treatments. It’s also important to carefully evaluate ethical issues, such as those pertaining to germline editing and the wider societal effects of gene editing technology.

However, further research is necessary before translating this novel technology to the clinic. Future surveys should evaluate it in greater numbers of animals and, lastly, in patients with respiratory disorders. In addition, further in vivo studies are required to examine the side effects of gene therapy-induced medication, potential immune responses triggered by viral delivery vectors, and more sensitive evaluations to reduce the risk of immunogenicity. The results of the first long-term report ultimately suggest that future preclinical trials should focus on improving transport and gene editing strategy in order to increase competence and increase the percentage of the targeted gene modifications.

## Conclusion and future directions

The current study sheds light on how the CRISPR/Cas system can combat drug resistance in respiratory disorders. It explores possibilities for treating diseases that were previously unexplored when it comes to understanding drug resistance. The development of CRISPR/Cas9-based gene editing technology has enabled precise and permanent targeting of mutations. The treatment of disorders can be significantly improved by changing the expression of genes linked to drug resistance. Although there are challenges, such as a few limitations, such as immunological reaction and delivery challenges or off-targeting, CRISPR/Cas9 allows researchers to identify and modify components linked to drug resistance with high accuracy. This level of precision is particularly valuable in addressing challenges like COPD, TB, and CF, where drug resistance has made treatment more difficult.

CRISPR/Cas9 gene editing enables incredibly tailored therapeutic interventions by precisely targeting genes linked to medication resistance in respiratory illnesses. Further, technological advances and lower costs have made CRISPR/Cas9 gene editing more accessible to researchers worldwide, facilitating collaboration and driving scientific discoveries. Despite its precision, CRISPR/Cas9 technology may cause genomic alterations off-target. Off-target effects are difficult to mitigate and require intensive experimental procedure assessment and modification. Additionally, delivering CRISPR/Cas9 components to the respiratory targeted cells is difficult. Clinical translation of CRISPR-based therapeutics requires overcoming cell-specific targeting and immune responses to delivery vectors. Further studies such as innovative delivery technologies like nanoparticles or viral vectors could improve respiratory illness CRISPR/Cas9 gene editing efficiency and specificity.

As genome editing technology based on CRISPR/Cas9 progresses from research to applications, it holds promising prospects for understanding and managing respiratory conditions. Therefore, using CRISPR/Cas9-based approaches will be a strategy in the era of personalized medicine for tackling the complexity of various respiratory diseases and overcoming treatment resistance in respiratory disorders, which aligns with the ultimate goals of precision medicine. Hence, further studies are required regarding ways of CRISPR/Cas9 gene editing to work in concert with traditional treatments, such immunotherapies or pharmacological drugs, may improve therapeutic results and overcoming drug resistance barriers. Future research should employ CRISPR/Cas9 to treat lung and other diseases such as aging and COVID-19.

## Data Availability

No datasets were generated or analysed during the current study.

## Abbreviations

AD: Adenocarcinoma
ANAPC5: Anaphase-promoting complex subunit 5
CDK1: Cyclin-dependent kinase 1
EGFR: Epidermal growth factor receptor
FGFR1: Fibroblast growth factor receptor 1
GFR-TKI: Growth factor receptor tyrosine kinase inhibitor
IGF1R: Insulin-like growth factor 1receptor
KEAP1: Kelch-like ECH-associated protein 1
LC: Lung cancer
LUAD: Lung adenocarcinoma
LUSC: Lung squamous cell carcinoma
MAPK: Mitogen-activated protein kinase
MDM4: Mouse double minute 4 homolog
MED1: Mediator Complex Subunit 1
NF-κB: Nuclear factor-kappa B
NSCLC: Non-small cell lung cancer
PI3Kβ: Phosphoinositide 3-kinase beta
PI3Kγ: Phosphoinositide 3-kinase gamma
PLK1: Polo-like kinase 1
PSMA6: Proteasome subunit alpha type-6
RSF1: Remodeling and spacing factor 1
